# Supplementary material for: Trends in Calcium Intake among the US Population: Results from the NHANES (1999–2018)
Source: Nutrients. 2024 Mar 2;16(5):726. doi: 10.3390/nu16050726 (PMC10934785; doi:10.3390/nu16050726)
Supplement: Supplementary file 1 [file nutrients-16-00726-s001.zip › nutrients-2851709-supplementary.pdf]

Table S1A Dietary calcium intake distribution (weighted) across DRIs among the US population, from 1999-2000 to 2017-2018

	Weighted Proportion of Population, % (95% CI)									
	1999-2000	2001-2002	2003-2004	2005-2006	2007-2008	2009-2010	2011-2012	2013-2014	2015-2016	2017-2018
**Men**										
<EAR	51.2(48.0,54.5)	47.2(44.4,50.0)	47.0(44.1,49.9)	42.8(39.7,45.8)	43.2(39.8,46.6)	38.9(37.2,40.7)	38.5(35.2,41.8)	41.8(39.4,44.2)	42.2(38.6,45.8)	40.2(37.7,42.7)
≥EAR, <RDA	12.9(11.4,14.5)	13.3(11.3,15.4)	14.5(12.8,16.3)	14.7(12.2,17.2)	15.8(15.0,16.7)	13.3(11.8,14.8)	14.2(11.9,16.5)	12.9(11.3,14.5)	14.3(12.4,16.2)	15.2(13.3,17.1)
≥RDA, ≤UL	33.9(31.2,36.7)	35.8(33.9,37.7)	35.0(32.8,37.2)	39.0(36.7,41.4)	37.6(34.5,40.6)	42.2(40.0,44.4)	42.8(40.3,45.3)	41.1(39.0,43.1)	39.7(35.9,43.4)	40.4(37.5,43.4)
>UL	1.90(1.24,2.55)	3.68(2.63,4.74)	3.47(2.57,4.37)	3.49(2.47,4.51)	3.43(2.24,4.63)	5.62(4.02,7.22)	4.46(3.22,5.70)	4.29(3.09,5.49)	3.83(2.51,5.16)	4.18(3.05,5.31)
**Women**										
<EAR	68.5(64.2,72.8)	65.7(63.8,67.6)	65.3(62.8,67.8)	61.9(57.5,66.3)	60.7(55.9,65.6)	56.8(54.9,58.8)	57.0(54.1,59.9)	59.9(57.5,62.2)	60.0(55.6,64.3)	58.9(55.6,62.2)
≥EAR, <RDA	10.8(8.04,13.5)	11.0(8.89,13.1)	11.0(10.0,11.9)	12.1(10.7,13.5)	13.4(11.0,15.7)	14.0(12.4,15.5)	13.8(12.1,15.5)	13.2(11.4,15.0)	14.1(12.3,15.9)	15.5(13.1,17.9)
≥RDA, ≤UL	19.9(17.3,22.5)	22.4(19.9,24.8)	22.7(19.7,25.6)	24.7(21.2,28.2)	24.8(21.6,28.0)	27.8(26.2,29.3)	28.2(25.5,31.0)	25.7(23.2,28.2)	24.9(21.5,28.3)	24.6(21.7,27.6)
>UL	0.81(0.39,1.23)	0.90(0.54,1.25)	1.08(0.45,1.72)	1.31(0.81,1.81)	1.08(0.45,1.71)	1.44(1.03,1.86)	0.97(0.30,1.63)	1.29(0.65,1.92)	1.06(0.50,1.61)	0.99(0.45,1.52)
**2-8 years old**										
<EAR	44.5(38.7,50.2)	35.0(30.2,39.9)	29.7(24.7,34.8)	35.0(31.9,38.2)	36.1(33.0,39.2)	29.9(27.0,32.7)	28.3(23.9,32.7)	34.5(30.8,38.2)	35.9(31.6,40.2)	33.6(29.1,38.0)
≥EAR, <RDA	18.8(15.2,22.5)	16.2(13.1,19.3)	19.0(14.5,23.6)	17.8(13.9,21.6)	18.2(14.8,21.5)	18.9(16.7,21.0)	18.1(15.4,20.8)	14.4(11.7,17.2)	19.1(15.9,22.3)	18.0(14.5,21.4)
≥RDA, ≤UL	36.5(30.6,42.4)	47.8(42.4,53.3)	50.2(43.8,56.5)	46.6(41.7,51.5)	45.3(41.6,49.1)	50.2(46.9,53.5)	52.6(48.1,57.1)	49.7(44.8,54.6)	44.3(40.6,48.1)	48.0(43.2,52.7)
>UL	0.22(0.00,0.57)	0.91(0.38,1.43)	1.05(0.27,1.83)	0.64(0.00,1.39)	0.42(0.04,0.80)	1.10(0.46,1.74)	1.02(0.13,1.91)	1.41(0.32,2.49)	0.69(0.00,1.48)	0.49(0.01,0.98)
**9-18 years old**										
<EAR	71.3(67.5,75.0)	66.0(61.1,70.9)	63.2(56.8,69.6)	64.9(59.7,70.1)	64.8(60.2,69.4)	59.6(54.8,64.3)	57.8(53.7,61.9)	62.3(58.0,66.6)	65.0(60.0,70.0)	64.5(58.9,70.2)
≥EAR, <RDA	7.72(5.96,9.47)	10.5(8.92,12.1)	9.81(7.17,12.5)	11.5(9.13,13.9)	12.0(9.29,14.7)	9.32(6.92,11.7)	10.8(7.50,14.0)	10.3(7.33,13.4)	10.1(7.39,12.9)	11.4(8.34,14.4)
≥RDA, ≤UL	20.4(17.5,23.3)	22.1(18.5,25.7)	25.9(19.8,32.1)	22.5(18.2,26.7)	22.1(19.1,25.1)	29.4(25.7,33.1)	31.0(26.7,35.2)	26.0(22.5,29.4)	23.7(19.5,27.9)	23.6(19.8,27.5)
>UL	0.66(0.13,1.20)	1.44(0.34,2.53)	1.03(0.28,1.79)	1.09(0.15,2.04)	1.10(0.53,1.67)	1.73(1.22,2.24)	0.46(0.00,1.02)	1.37(0.09,2.65)	1.11(0.41,1.81)	0.44(0.00,0.89)
**19-59 years old**										
<EAR	56.2(51.3,61.0)	52.9(49.9,55.9)	53.7(50.4,56.9)	47.6(43.2,52.0)	47.1(42.4,51.8)	42.9(41.6,44.2)	43.7(40.6,46.7)	46.3(43.8,48.9)	44.9(40.6,49.1)	43.5(41.2,45.9)
≥EAR, <RDA	12.4(10.0,14.7)	12.9(11.1,14.8)	13.2(11.6,14.7)	13.5(12.1,15.0)	15.1(13.0,17.2)	15.0(14.0,15.9)	14.8(12.5,17.1)	14.3(12.3,16.3)	15.5(13.9,17.1)	16.7(14.6,18.8)
≥RDA, ≤UL	29.7(26.3,33.0)	31.6(28.5,34.6)	30.2(26.8,33.7)	35.9(32.5,39.3)	34.8(30.9,38.8)	38.2(36.6,39.7)	38.5(35.9,41.0)	36.1(33.4,38.8)	36.9(32.9,40.9)	36.6(33.9,39.4)
>UL	1.78(1.03,2.52)	2.63(1.69,3.57)	2.92(2.16,3.68)	3.02(1.98,4.05)	2.92(2.01,3.83)	3.95(2.70,5.21)	3.09(2.22,3.95)	3.23(2.21,4.26)	2.75(1.74,3.76)	3.12(1.84,4.40)
**60 years or older**										
<EAR	74.3(71.4,77.3)	75.7(72.3,79.1)	73.2(69.6,76.8)	67.9(63.1,72.7)	68.9(64.4,73.3)	65.1(62.5,67.7)	63.6(59.2,68.0)	63.6(60.3,66.9)	66.1(60.8,71.4)	63.0(57.3,68.6)
≥EAR, <RDA	8.96(6.62,11.3)	8.15(6.18,10.1)	10.3(7.95,12.6)	11.9(9.44,14.3)	12.6(10.3,14.8)	10.0(8.28,11.8)	11.8(9.93,13.7)	10.7(8.50,12.9)	11.2(8.57,13.9)	13.3(10.2,16.4)
≥RDA, ≤UL	15.5(13.9,17.1)	13.6(10.8,16.4)	14.8(12.1,17.4)	17.9(15.0,20.8)	16.8(13.8,19.8)	20.3(18.1,22.5)	20.7(17.1,24.3)	22.7(19.8,25.5)	19.6(16.6,22.5)	20.5(15.7,25.4)
>UL	1.17(0.00,2.51)	2.51(1.23,3.79)	1.79(1.08,2.50)	2.31(1.12,3.49)	1.74(0.65,2.82)	4.57(3.16,5.98)	3.90(2.93,4.87)	3.03(1.91,4.14)	3.09(1.38,4.80)	3.19(2.06,4.31)
**Mexican American**										
<EAR	60.7(57.7,63.7)	52.9(48.6,57.2)	50.2(44.3,56.0)	51.7(47.6,55.8)	51.8(48.8,54.9)	49.2(44.1,54.3)	40.6(37.4,43.8)	46.3(42.0,50.5)	49.0(45.9,52.0)	44.5(40.8,48.2)
≥EAR, <RDA	13.1(10.9,15.3)	14.7(11.5,18.0)	13.0(10.1,15.9)	12.8(10.7,14.8)	14.2(11.5,16.9)	13.6(11.3,16.0)	14.7(12.2,17.2)	12.6(10.1,15.2)	14.7(12.0,17.5)	16.0(12.2,19.8)
≥RDA, ≤UL	24.7(21.3,28.2)	30.9(28.9,33.0)	35.8(30.3,41.2)	34.0(30.0,38.1)	32.4(29.6,35.1)	34.3(29.9,38.7)	42.1(39.3,44.8)	38.7(35.2,42.1)	33.6(30.4,36.9)	37.4(33.7,41.2)
>UL	1.50(0.96,2.05)	1.47(0.52,2.43)	1.07(0.59,1.55)	1.51(0.56,2.46)	1.62(0.42,2.83)	2.93(1.97,3.89)	2.61(0.52,4.69)	2.43(0.94,3.92)	2.63(1.83,3.43)	2.09(0.90,3.28)
**Other Hispanic**										
<EAR	63.8(58.2,69.3)	56.8(50.9,62.8)	66.9(59.7,74.1)	55.0(43.1,66.9)	50.0(45.8,54.2)	47.8(43.4,52.2)	51.5(47.1,56.0)	51.2(45.5,56.9)	52.7(47.9,57.6)	50.9(44.6,57.1)
≥EAR, <RDA	13.5(8.07,19.0)	17.1(11.3,22.9)	8.80(4.54,13.1)	12.3(7.19,17.5)	12.1(9.05,15.1)	15.2(11.0,19.3)	13.8(8.45,19.2)	14.4(11.4,17.4)	14.4(12.2,16.7)	15.8(13.7,17.9)
≥RDA, ≤UL	21.6(16.0,27.2)	24.2(19.8,28.7)	23.1(17.7,28.5)	31.7(20.1,43.2)	36.0(32.4,39.6)	35.4(31.7,39.1)	33.1(27.6,38.6)	32.2(27.0,37.4)	31.5(25.6,37.4)	32.1(25.5,38.7)
>UL	1.10(0.00,2.51)	1.81(0.54,3.08)	1.20(0.00,2.75)	0.97(0.00,2.09)	1.97(1.08,2.86)	1.63(0.57,2.68)	1.59(0.35,2.83)	2.24(0.75,3.73)	1.30(0.00,2.66)	1.26(0.19,2.34)

	Weighted Proportion of Population, % (95% CI)									
	**1999-2000**	**2001-2002**	**2003-2004**	**2005-2006**	**2007-2008**	**2009-2010**	**2011-2012**	**2013-2014**	**2015-2016**	**2017-2018**
**Non-Hispanic White**										
<EAR	56.2(51.0,61.4)	53.8(51.1,56.4)	53.5(50.7,56.3)	49.8(45.8,53.8)	49.9(44.8,55.0)	45.5(42.9,48.1)	45.3(42.0,48.6)	48.8(46.2,51.4)	48.4(43.9,52.9)	47.9(44.4,51.4)
≥EAR, <RDA	12.1(10.4,13.7)	12.0(10.6,13.5)	13.2(11.8,14.6)	14.0(11.7,16.2)	15.2(13.3,17.1)	13.6(11.8,15.3)	14.4(11.8,17.0)	13.3(11.6,15.0)	14.2(12.0,16.3)	16.0(13.6,18.4)
≥RDA, ≤UL	30.3(26.3,34.2)	31.4(29.1,33.7)	30.5(27.9,33.2)	33.3(30.6,36.1)	32.2(28.6,35.8)	36.6(34.4,38.9)	37.0(34.0,40.1)	34.5(31.3,37.7)	34.5(30.8,38.2)	33.0(29.9,36.1)
>UL	1.48(1.07,1.90)	2.81(1.94,3.68)	2.81(2.14,3.48)	2.94(2.13,3.75)	2.65(1.80,3.51)	4.28(3.01,5.56)	3.25(2.33,4.18)	3.40(2.31,4.48)	2.97(1.85,4.09)	3.09(2.05,4.14)
**Non-Hispanic Black**										
<EAR	73.5(69.6,77.4)	72.0(68.2,75.9)	70.0(66.4,73.6)	65.1(61.4,68.7)	62.4(58.4,66.4)	58.3(55.5,61.0)	58.5(54.1,62.8)	61.6(58.1,65.0)	62.9(58.4,67.4)	59.8(55.8,63.9)
≥EAR, <RDA	10.3(7.95,12.6)	9.67(6.66,12.7)	11.0(9.36,12.6)	10.5(8.48,12.5)	13.7(11.8,15.6)	13.3(11.1,15.5)	12.3(10.6,13.9)	10.4(9.05,11.8)	13.4(10.8,16.0)	12.6(10.1,15.1)
≥RDA, ≤UL	15.8(13.2,18.3)	18.0(13.7,22.3)	18.4(16.3,20.4)	23.7(20.7,26.7)	22.8(19.2,26.4)	26.8(22.9,30.7)	27.7(23.8,31.6)	26.5(23.8,29.1)	22.6(18.9,26.4)	25.8(22.8,28.9)
>UL	0.41(0.00,0.94)	0.29(0.02,0.56)	0.64(0.06,1.22)	0.77(0.09,1.44)	1.13(0.48,1.78)	1.59(1.01,2.18)	1.57(0.75,2.40)	1.53(0.67,2.40)	1.09(0.38,1.80)	1.74(1.01,2.46)
**Other Race or Ethnicity [a]**										
<EAR	74.7(67.2,82.2)	69.2(61.5,76.9)	66.7(58.0,75.4)	59.7(53.2,66.2)	63.5(56.3,70.7)	54.5(48.2,60.7)	57.5(53.6,61.3)	57.7(51.1,64.4)	57.5(50.6,64.4)	54.2(49.6,58.9)
≥EAR, <RDA	7.95(3.56,12.3)	9.12(5.81,12.4)	12.1(9.18,15.0)	13.7(8.77,18.5)	11.4(7.09,15.7)	13.5(8.94,18.1)	13.1(10.2,15.9)	14.3(8.97,19.6)	14.4(10.3,18.6)	13.5(11.7,15.3)
≥RDA, ≤UL	15.7(9.52,22.0)	20.8(14.7,27.0)	20.0(12.5,27.6)	25.6(17.4,33.7)	24.8(18.4,31.3)	30.8(25.6,36.1)	28.5(24.3,32.7)	27.3(23.2,31.4)	27.1(21.6,32.6)	30.6(26.1,35.1)
>UL	1.63(0.00,3.60)	0.84(0.00,2.15)	1.15(0.00,2.51)	1.07(0.00,2.47)	0.26(0.00,0.80)	1.21(0.02,2.41)	0.98(0.29,1.67)	0.68(0.07,1.29)	0.97(0.28,1.66)	1.66(0.40,2.92)
**Overall**										
<EAR	60.1(56.7,63.6)	56.7(54.7,58.7)	56.3(54.0,58.6)	52.6(49.4,55.8)	52.3(48.5,56.2)	48.1(46.8,49.4)	47.9(45.3,50.4)	50.9(49.0,52.9)	51.3(47.5,55.1)	49.8(47.2,52.4)
≥EAR, <RDA	11.8(10.3,13.4)	12.1(10.8,13.5)	12.7(11.7,13.8)	13.4(12.0,14.7)	14.5(13.3,15.8)	13.6(12.6,14.7)	14.0(12.3,15.8)	13.0(11.8,14.3)	14.2(13.0,15.4)	15.3(13.7,17.0)
≥RDA, ≤UL	26.7(24.3,29.1)	28.9(26.9,31.0)	28.7(26.5,31.0)	31.7(29.3,34.1)	30.9(28.0,33.8)	34.8(33.4,36.1)	35.4(33.3,37.6)	33.3(31.4,35.2)	32.1(28.9,35.3)	32.3(30.2,34.4)
>UL	1.34(0.99,1.69)	2.25(1.62,2.89)	2.26(1.64,2.87)	2.37(1.85,2.89)	2.21(1.54,2.88)	3.48(2.64,4.31)	2.69(2.16,3.23)	2.77(2.09,3.44)	2.41(1.68,3.14)	2.54(1.87,3.21)

Abbreviations: CI, confidence interval; EAR, Estimated Average Requirement; RDA, Recommended Dietary Allowance; UL, Tolerable Upper Intake Level.

[a] Included multi-racial/ethnic groups.

Table S1B Prevalence of calcium-containing dietary supplement use among the US population with dietary calcium intake lower than their EARs, from 1999-2000 to 2017-2018

	Weighted Proportion of Population, % (95% CI)									
	**1999-2000**	**2001-2002**	**2003-2004**	**2005-2006**	**2007-2008**	**2009-2010**	**2011-2012**	**2013-2014**	**2015-2016**	**2017-2018**
**Men**										
no supplement	69.3(64.5,74.1)	67.0(63.7,70.2)	64.1(60.2,68.0)	63.9(59.1,68.7)	70.8(66.7,74.8)	69.1(66.1,72.0)	72.1(67.5,76.7)	68.9(65.4,72.4)	72.0(68.8,75.2)	73.4(68.7,78.0)
with supplement	30.7(25.9,35.5)	33.0(29.8,36.3)	35.9(32.0,39.8)	36.1(31.3,40.9)	29.2(25.2,33.3)	30.9(28.0,33.9)	27.9(23.32,32.5)	31.1(27.6,34.6)	28.0(24.8,31.2)	26.6(22.0,31.3)
**Women**										
no supplement	58.5(53.3,63.6)	52.6(49.6,55.6)	53.9(49.4,58.4)	51.3(48.2,54.3)	59.0(56.7,61.2)	59.7(55.1,64.4)	62.2(59.7,64.8)	62.1(58.7,65.6)	64.2(60.4,68.0)	62.4(59.3,65.5)
with supplement	41.5(36.4,46.7)	47.4(44.4,50.4)	46.1(41.6,50.6)	48.7(45.7,51.8)	41.0(38.8,43.3)	40.3(35.6,44.9)	37.8(35.2,40.3)	37.9(34.4,41.3)	35.8(32.0,39.6)	37.6(34.5,40.7)
**2-8 years old**										
no supplement	82.9(78.4,87.5)	74.5(68.6,80.5)	80.0(71.2,88.9)	77.4(70.3,84.4)	76.4(66.9,85.9)	85.1(80.8,89.4)	85.7(78.5,92.8)	91.0(88.3,93.7)	90.6(86.4,94.8)	91.9(88.7,95.1)
with supplement	17.1(12.5,21.6)	25.5(19.5,31.4)	20.0(11.1,28.8)	22.6(15.6,29.7)	23.6(14.1,33.1)	14.9(10.6,19.2)	14.3(7.18,21.5)	9.00(6.28,11.7)	9.38(5.20,13.6)	8.09(4.92,11.3)
**9-18 years old**										
no supplement	82.6(79.8,85.4)	80.0(75.4,84.7)	83.8(80.3,87.3)	79.4(76.3,82.5)	82.4(77.5,87.3)	84.9(80.5,89.4)	86.2(82.8,89.6)	87.0(83.5,90.5)	88.5(84.4,92.7)	85.8(81.1,90.5)
with supplement	17.4(14.6,20.2)	20.0(15.3,24.6)	16.2(12.7,19.7)	20.6(17.5,23.7)	17.6(12.7,22.5)	15.1(10.6,19.5)	13.8(10.4,17.2)	13.0(9.46,16.5)	11.5(7.30,15.6)	14.2(9.54,18.9)
**19-59 years old**										
no supplement	60.1(54.9,65.2)	56.5(53.3,59.8)	56.6(52.0,61.2)	52.4(48.5,56.3)	64.2(60.9,67.6)	62.3(56.4,68.2)	66.4(62.5,70.2)	63.4(60.4,66.5)	68.4(63.4,73.4)	67.7(64.1,71.4)
with supplement	39.9(34.8,45.1)	43.5(40.2,46.7)	43.4(38.8,48.0)	47.6(43.7,51.5)	35.8(32.4,39.1)	37.7(31.8,43.6)	33.6(29.8,37.5)	36.6(33.5,39.6)	31.6(26.6,36.6)	32.3(28.6,35.9)
**60 years or older**										
no supplement	45.8(39.9,51.7)	40.3(36.0,44.5)	38.2(33.7,42.7)	41.2(37.1,45.4)	44.5(39.7,49.3)	45.3(40.8,49.8)	48.8(45.5,52.1)	46.6(42.8,50.3)	46.6(41.8,51.3)	47.6(42.1,53.0)
with supplement	54.2(48.3,60.1)	59.7(55.5,64.0)	61.8(57.3,66.3)	58.8(54.6,62.9)	55.5(50.7,60.3)	54.7(50.2,59.2)	51.2(47.9,54.5)	53.4(49.7,57.2)	53.4(48.7,58.2)	52.4(47.0,57.9)
**Mexican American**										
no supplement	75.7(73.4,78.0)	76.6(73.5,79.7)	72.5(65.9,79.1)	77.8(71.3,84.2)	82.9(76.9,88.9)	76.7(72.8,80.7)	80.1(74.8,85.5)	81.6(77.7,85.5)	78.3(76.5,80.0)	80.6(77.1,84.2)
with supplement	24.3(22.0,26.6)	23.4(20.3,26.5)	27.5(20.9,34.1)	22.2(15.80,28.7)	17.1(11.1,23.1)	23.3(19.3,27.2)	19.9(14.5,25.2)	18.4(14.5,22.3)	21.7(20.0,23.5)	19.4(15.8,22.9)
**Other Hispanic**										
no supplement	74.5(69.9,79.1)	73.3(67.2,79.3)	67.0(58.8,75.2)	78.0(69.7,86.4)	77.9(71.9,83.9)	79.3(74.2,84.4)	72.8(62.5,83.0)	78.4(73.6,83.2)	79.6(74.8,84.4)	70.2(64.1,76.2)
with supplement	25.5(20.9,30.1)	26.7(20.7,32.8)	33.0(24.8,41.2)	22.0(13.6,30.3)	22.1(16.1,28.1)	20.7(15.6,25.8)	27.2(17.0,37.5)	21.6(16.8,26.4)	20.4(15.6,25.2)	29.8(23.8,35.9)
**Non-Hispanic White**										
no supplement	56.3(51.1,61.5)	49.8(46.6,52.9)	50.5(46.4,54.6)	47.6(44.5,50.8)	56.2(52.9,59.5)	56.0(51.6,60.5)	60.2(56.5,63.8)	56.6(54.9,58.3)	60.9(57.0,64.9)	61.3(56.6,66.0)
with supplement	43.7(38.5,48.9)	50.2(47.1,53.4)	49.5(45.4,53.6)	52.4(49.2,55.5)	43.8(40.5,47.1)	44.0(39.5,48.4)	39.8(36.2,43.5)	43.4(41.7,45.1)	39.1(35.1,43.0)	38.7(34.0,43.4)
**Non-Hispanic Black**										
no supplement	79.0(75.6,82.3)	77.0(73.1,81.0)	81.0(77.9,84.1)	75.1(70.7,79.6)	77.3(74.3,80.2)	77.2(74.3,80.0)	78.0(75.4,80.7)	79.8(74.5,85.2)	79.4(75.1,83.6)	76.8(72.2,81.4)
with supplement	21.0(17.7,24.4)	23.0(19.0,26.9)	19.0(15.9,22.1)	24.9(20.4,29.3)	22.7(19.8,25.7)	22.8(20.0,25.7)	22.0(19.3,24.6)	20.2(14.8,25.5)	20.6(16.4,24.9)	23.2(18.6,27.8)
**Other Race or Ethnicity** [a]										
no supplement	64.1(53.5,74.7)	70.1(60.4,79.7)	60.5(48.2,72.7)	59.1(44.7,73.6)	68.2(56.7,79.6)	68.2(59.4,76.9)	68.2(61.5,74.8)	68.8(65.3,72.3)	67.5(61.3,73.8)	67.4(59.4,75.4)
with supplement	35.9(25.3,46.5)	29.9(20.3,39.6)	39.5(27.3,51.8)	40.9(26.4,55.3)	31.8(20.4,43.3)	31.8(23.1,40.6)	31.8(25.2,38.5)	31.2(27.7,34.7)	32.5(26.2,38.7)	32.6(24.6,40.6)
**Overall**										
no supplement	63.0(59.1,66.9)	58.4(55.7,61.1)	58.1(54.3,61.9)	56.3(53.5,59.1)	63.6(61.0,66.3)	63.4(59.7,67.1)	66.2(63.1,69.2)	64.9(62.4,67.4)	67.3(64.3,70.4)	66.7(64.3,69.1)
with supplement	37.0(33.1,40.9)	41.6(38.9,44.3)	41.9(38.1,45.7)	43.7(40.9,46.5)	36.4(33.7,39.0)	36.6(32.9,40.3)	33.8(30.8,36.9)	35.1(32.6,37.6)	32.7(29.6,35.7)	33.3(30.9,35.7)

Abbreviations: CI, confidence interval; EAR, Estimated Average Requirement.

[a] Included multi-racial/ethnic groups.

Table S2 Dietary and dietary supplement calcium intake distribution (weighted) across DRIs among the US population, from 2007-2008 to 2017-2018

	Weighted Proportion of Population, % (95% CI)					
	**2007-2008**	**2009-2010**	**2011-2012**	**2013-2014**	**2015-2016**	**2017-2018**
**Men**						
<EAR	39.2(35.7,42.7)	34.6(33.3,36.0)	34.2(31.2,37.3)	37.4(34.7,40.2)	37.9(34.4,41.4)	37.7(35.2,40.1)
≥EAR, <RDA	13.6(12.5,14.7)	12.6(11.3,14.0)	13.8(11.6,16.1)	12.8(11.0,14.7)	13.9(12.3,15.6)	14.0(12.2,15.8)
≥RDA, ≤UL	41.6(38.0,45.3)	45.0(43.3,46.6)	46.0(43.7,48.4)	43.6(40.8,46.4)	42.8(39.3,46.3)	42.1(38.8,45.4)
>UL	5.58(4.18,6.98)	7.79(6.42,9.16)	5.94(4.47,7.40)	6.13(4.48,7.79)	5.39(3.82,6.97)	6.26(4.27,8.24)
**Women**						
<EAR	47.9(43.1,52.8)	44.6(42.4,46.7)	46.5(43.1,49.8)	48.6(46.4,50.7)	50.6(45.8,55.5)	50.1(47.0,53.3)
≥EAR, <RDA	12.2(10.3,14.0)	12.9(11.3,14.6)	13.7(12.1,15.2)	13.2(11.9,14.5)	14.7(12.0,17.3)	14.4(12.3,16.5)
≥RDA, ≤UL	34.2(30.8,37.5)	36.3(34.2,38.3)	34.9(32.0,37.7)	33.0(30.4,35.6)	30.7(26.0,35.3)	31.4(28.1,34.8)
>UL	5.77(3.93,7.61)	6.23(5.40,7.07)	4.97(3.76,6.19)	5.23(3.88,6.59)	4.02(2.88,5.15)	4.03(3.05,5.02)
**2-8 years old**						
<EAR	34.7(31.6,37.7)	28.9(26.4,31.4)	26.9(22.7,31.2)	33.8(30.5,37.2)	35.6(31.2,40.1)	33.2(29.0,37.5)
≥EAR, <RDA	18.0(14.6,21.4)	17.7(14.9,20.5)	18.7(15.9,21.4)	14.5(11.8,17.2)	18.9(15.7,22.0)	18.1(14.5,21.7)
≥RDA, ≤UL	46.9(43.6,50.3)	52.3(48.7,55.9)	53.1(49.0,57.2)	50.3(45.4,55.1)	44.7(41.2,48.3)	48.2(43.6,52.8)
>UL	0.42(0.04,0.80)	1.10(0.46,1.74)	1.27(0.35,2.19)	1.41(0.32,2.49)	0.80(0.00,1.60)	0.49(0.01,0.98)
**9-18 years old**						
<EAR	62.6(57.9,67.3)	58.3(53.8,62.8)	56.2(51.5,60.8)	61.0(56.7,65.4)	63.9(58.5,69.3)	64.0(58.3,69.8)
≥EAR, <RDA	12.2(9.85,14.5)	9.54(7.43,11.7)	11.5(8.25,14.7)	11.0(8.11,13.9)	10.6(7.59,13.7)	11.3(8.38,14.2)
≥RDA, ≤UL	24.0(20.7,27.2)	30.4(26.7,34.2)	31.7(27.0,36.4)	26.6(23.2,30.1)	24.2(19.7,28.6)	24.1(20.1,28.1)
>UL	1.22(0.68,1.76)	1.73(1.22,2.24)	0.65(0.00,1.31)	1.37(0.09,2.65)	1.27(0.41,2.13)	0.59(0.07,1.10)
**19-59 years old**						
<EAR	40.2(35.8,44.5)	35.7(33.7,37.7)	37.9(34.3,41.6)	39.4(36.8,42.0)	40.1(35.6,44.6)	39.3(36.4,42.1)
≥EAR, <RDA	12.4(11.1,13.6)	13.5(11.9,15.1)	13.7(11.8,15.7)	14.0(12.6,15.5)	14.7(13.1,16.2)	14.7(12.9,16.4)
≥RDA, ≤UL	41.3(36.8,45.8)	43.7(41.5,45.8)	43.4(40.9,45.9)	41.1(38.2,44.1)	40.5(36.3,44.8)	40.7(37.9,43.5)
>UL	6.23(4.52,7.94)	7.21(5.97,8.46)	5.01(3.52,6.50)	5.42(4.06,6.78)	4.69(3.23,6.15)	5.35(3.86,6.85)
**60 years or older**						
<EAR	45.6(40.0,51.2)	43.8(41.7,46.0)	44.0(40.0,47.9)	45.4(43.1,47.7)	47.1(42.9,51.2)	48.2(42.4,53.9)
≥EAR, <RDA	12.1(9.30,15.0)	10.6(9.49,11.6)	12.9(10.5,15.2)	10.9(8.96,12.8)	13.7(9.85,17.6)	13.2(10.8,15.7)
≥RDA, ≤UL	31.9(27.9,35.9)	32.2(29.7,34.7)	31.0(27.6,34.3)	32.5(28.7,36.2)	30.6(25.8,35.4)	29.4(23.4,35.5)
>UL	10.4(7.36,13.4)	13.4(11.6,15.2)	12.2(9.19,15.2)	11.3(9.13,13.5)	8.55(6.41,10.7)	9.17(7.36,11.0)
**Mexican American**						
<EAR	48.1(45.0,51.2)	44.8(39.8,49.9)	36.9(34.2,39.5)	42.9(37.8,48.0)	45.3(42.3,48.3)	41.8(37.9,45.8)
≥EAR, <RDA	14.6(12.3,16.9)	13.9(11.4,16.5)	15.2(12.6,17.9)	13.0(9.76,16.2)	15.3(12.7,17.9)	15.2(11.3,19.1)
≥RDA, ≤UL	34.8(31.8,37.7)	37.2(33.0,41.4)	44.7(41.7,47.7)	40.7(37.4,44.0)	36.2(32.9,39.5)	39.6(36.4,42.7)
>UL	2.56(1.25,3.88)	4.02(2.79,5.25)	3.24(1.14,5.33)	3.43(1.77,5.09)	3.19(2.30,4.09)	3.38(1.85,4.91)
**Other Hispanic**						
<EAR	46.1(42.3,49.8)	43.9(39.6,48.2)	46.3(41.5,51.0)	47.2(41.4,52.9)	49.3(44.8,53.8)	46.3(39.4,53.2)
≥EAR, <RDA	11.9(8.55,15.2)	13.5(9.30,17.7)	14.5(10.3,18.7)	14.8(11.4,18.2)	15.0(12.3,17.6)	16.3(13.6,19.0)
≥RDA, ≤UL	38.9(35.7,42.0)	38.8(36.0,41.7)	35.8(31.8,39.7)	35.3(30.3,40.2)	33.4(27.7,39.0)	35.4(28.2,42.6)
>UL	3.18(1.62,4.75)	3.74(2.34,5.14)	3.46(1.81,5.11)	2.80(1.27,4.33)	2.39(0.22,4.55)	1.98(0.53,3.43)
**Non-Hispanic White**						
<EAR	39.5(34.3,44.8)	35.3(33.4,37.1)	36.7(33.5,39.9)	39.2(36.3,42.1)	40.2(36.1,44.4)	40.9(38.0,43.8)
≥EAR, <RDA	12.7(11.3,14.0)	12.4(10.4,14.4)	13.6(11.3,15.8)	13.1(11.7,14.4)	14.4(12.3,16.6)	14.3(12.6,16.0)
≥RDA, ≤UL	40.8(36.4,45.1)	43.5(41.1,45.9)	42.8(40.0,45.7)	40.4(37.0,43.8)	39.1(34.8,43.5)	38.3(35.4,41.2)
>UL	7.04(5.06,9.02)	8.85(7.52,10.2)	6.90(5.50,8.29)	7.35(5.66,9.04)	6.20(4.60,7.81)	6.47(5.07,7.87)
**Non-Hispanic Black**						
<EAR	58.5(55.2,61.8)	53.6(50.3,56.9)	53.1(48.2,58.0)	56.0(52.8,59.3)	59.4(54.7,64.2)	56.2(52.4,60.1)
≥EAR, <RDA	12.8(10.9,14.8)	13.5(12.1,14.9)	12.9(11.1,14.8)	11.2(9.58,12.8)	12.8(10.0,15.6)	12.6(10.4,14.8)
≥RDA, ≤UL	26.1(23.0,29.2)	30.3(26.6,34.0)	31.2(27.1,35.2)	30.8(28.3,33.3)	26.3(22.8,29.8)	28.2(25.9,30.4)
>UL	2.53(1.63,3.43)	2.61(1.62,3.60)	2.80(1.47,4.12)	1.96(1.12,2.79)	1.48(0.68,2.27)	2.97(1.59,4.36)
**Other Race or Ethnicity**						
<EAR	53.2(43.8,62.7)	47.9(40.7,55.0)	49.6(46.2,53.0)	51.0(45.0,57.0)	48.5(41.7,55.4)	49.0(45.5,52.5)
≥EAR, <RDA	13.1(8.78,17.5)	13.0(9.21,16.8)	14.1(10.7,17.5)	13.9(9.31,18.5)	13.9(11.6,16.2)	13.1(11.1,15.1)
≥RDA, ≤UL	30.0(21.4,38.6)	35.5(29.4,41.6)	33.9(30.2,37.6)	31.7(27.5,35.9)	35.4(29.5,41.3)	34.1(30.5,37.8)
>UL	3.66(0.15,7.16)	3.63(0.92,6.35)	2.42(0.90,3.94)	3.42(1.55,5.29)	2.18(1.04,3.32)	3.78(0.89,6.67)
**Overall**						
<EAR	43.7(39.8,47.7)	39.7(38.4,41.0)	40.4(37.7,43.1)	43.1(41.1,45.0)	44.4(40.5,48.4)	44.1(41.6,46.5)
≥EAR, <RDA	12.9(11.9,13.8)	12.8(11.5,14.1)	13.8(12.2,15.3)	13.0(11.8,14.2)	14.3(13.0,15.7)	14.2(12.9,15.5)
≥RDA, ≤UL	37.7(34.5,41.0)	40.5(38.8,42.2)	40.4(38.3,42.5)	38.2(36.0,40.4)	36.6(33.0,40.2)	36.6(34.6,38.6)
>UL	5.68(4.27,7.09)	6.99(6.10,7.88)	5.45(4.43,6.47)	5.68(4.53,6.82)	4.69(3.51,5.87)	5.12(4.22,6.01)

Abbreviation: CI, confidence interval; EAR, Estimated Average Requirement; RDA, Recommended Dietary Allowance; UL, Tolerable Upper Intake Level.
